# Psychomotor development in very and extremely low-birth-weight preterm children: Could it be predicted by early motor milestones and perinatal complications?

**DOI:** 10.3934/Neuroscience.2022011

**Published:** 2022-04-28

**Authors:** Cristina Fernandez-Baizan, Leticia Alcantara-Canabal, Marta Mendez, Gonzalo Solis

**Affiliations:** 1 Department of Psychology. University of Oviedo, Oviedo, Spain; 2 Neuroscience Institute of Principado de Asturias (INEUROPA), Oviedo, Spain; 3 Instituto de Investigación Sanitaria del Principado de Asturias (ISPA), Oviedo, Spain; 4 Primary Care Center, Paulino Prieto, Sanitary Area IV, Oviedo, Spain; 5 Pediatric Clinic Area, Neonatology, Hospital Universitario Central de Asturias (HUCA), Oviedo, Spain

**Keywords:** preterm, psychomotor development, perinatal risk, motor milestones, low birth weight

## Abstract

Preterm-born children are at risk of slower psychomotor development. This risk may be associated with low birth weight and other perinatal factors and morbidities.

We aimed to assess psychomotor development in school-aged preterm children, and to determine whether some early motor and perinatal variables could be related to and/or predict the later motor achievements.

Parents of 54 very low-birth-weight preterm, 24 extremely low-birth-weight preterm and 96 control children completed the Movement Assessment Battery for Children (MABC-2-C) checklist and were interviewed about the motor milestones of their children.

Significant differences were found between the preterm and control groups in the MABC-2-C results. MABC-2-C outcomes were significantly predicted by the age of crawling, the use of steroids, mechanical ventilation and intraventricular hemorrhage (IVH).

The use of screening tools may allow the rapid identification of psychomotor development delays. The presence of some perinatal risk factors and some motor milestone attainments could be related to motor development in the later childhood of preterm children.

## 1. Introduction

Preterm-born children are at risk of a wide variety of developmental issues: neuropsychological alterations, behavioral problems, academic difficulties and motor alterations [1]. The motor abilities found to be impaired in this population include fine and gross motor skills, balance skills, ball skills and manual dexterity [2]. These could be compatible with the diagnosis of developmental coordination disorder (DCD) [3], which is related to difficulties with motor coordination, clumsiness, slowness or imprecision in motor tasks. DCD is more prevalent in low-birth-weight and very preterm populations, varying between 1.7% and 6% in school-aged children [4]. Besides, being born very or moderately preterm has been identified as a risk factor for DCD [5].

A great deal of previous studies have employed the Movement Assessment Battery for Children-2 (MABC-2) for assessing preterm populations [6], and for DCD diagnosis [7]. Application of the entire battery in a regular consultation is not always feasible because it requires a lot of time and effort. Therefore, the use of a motor checklist as a screening method could have many benefits. The MABC-2 checklist is practical for the quick screening of daily motor impairments in children [7].

Regarding the preterm population, several motor symptoms can be detected in early stages of development [8], as well as the delayed attainment of some motor milestones [9]. Preterm-born children are at risk of motor difficulties, and these difficulties are more common in extremely low-birth-weight (ELBW) preterm children [2]. Likewise, there are some other early risk factors that could affect the subsequent development of preterm children [10]. However, there is no consensus in existing literature about which factors could predict poor motor outcomes. For this reason, it is very important to study the impact of early risk factors on later motor development.

Hence, the main aim of the current study was to assess motor development in ELBW and very low-birth-weight (VLBW) preterm and normally developing children aged 5 to 7 years by employing a screening method: the MABC-2 checklist. Our hypothesis was that preterm children would score lower than control children on the overall scale and on each subscale. Besides, we aimed to determine whether the current motor development of preterm children could be associated with some early developmental factors, such as motor milestone attainments and perinatal risk factors.

## 2. Material and methods

### 2.1. Study design and participants

The study conducted was observational, descriptive and cross-sectional. Preterm children (5–7 years old, chronological age) were recruited from a cohort of neonates who were born before 37 weeks of gestation, had a birthweight under 1500 g and were admitted to the neonatal intensive care unit (NICU) of the Hospital Universitario Central de Asturias (Oviedo, Spain) between January 2009 and December 2011. The inclusion criteria were a gestational age of ≤37 weeks and a birth weight of ≤1500 g. The exclusion criteria were death, no follow-up, preterm children with a diagnosis of malformations and/or congenital anomalies that led to evident neurological alterations, a diagnosis of cerebral palsy or an intelligence quotient (IQ) of ≤70, as determined by the Reynolds Intellectual Screening Test (RIST) test [11]. Control children (5–7 years old) born at term (>37 weeks) were recruited from schools, primary care centers and hospitals in Oviedo, Spain. The initial sample was composed of 147 preterm children and 155 control children. The final sample was composed of 78 preterm children (54 with VLBW and 24 with ELBW) and 96 control children. Sociodemographic and milestone achievements of the preterm and control groups are shown in Table 1. The presence of perinatal risk factors in the preterm sample is shown in Table 2. The study was conducted in accordance with the Helsinki declaration for research in human subjects and approved by the regional ethics committee (Comité de Ética de la Investigación del Principado de Asturias).

**Table 1.** Sociodemographic and milestone achievements and IQ descriptive data on the ELBW and VLBW preterm and control groups.

		ELBW preterm n=24 N (%)	VLBW preterm n=54	Controls n=96
Age	5	11 (45.8%)	15 (27.8%)	34 (35.4%)
	6	5 (20.8%)	19 (35.2%)	32 (33.3%)
	7	8 (33.3%)	20 (37%)	30 (31.3%)
Gender	Males	13 (54.2%)	32 (59.3%)	45 (46.9%)
	Females	11 (45.8%)	22 (40.7%)	51 (53.1%)
Maternal educational level	Bachelor's degree	12 (52.2%)	23 (42.6%)	35 (36.5%)
	Technical	6 (26.1%)	21 (38.9%)	13 (13.5%)
	Secondary	5 (21.7%)	6 (11.1%)	5 (5.2%)
	Primary	0 (0.0 %)	4 (7.4%)	0 (0.0 %)
	Not reported	1 (0.0%)	0 (0.0%)	43 (44,7%)
Laterality	Right	19 (86.4%)	44 (86.3%)	85 (88.5%)
	Left	1 (4.5%)	6 (11.8%)	10 (10.4%)
	Both	2 (9.1%)	1 (2.0%)	1 (1.0%)
	Mean (SD)
RIST (Standardized scores)	91.23 (16.31)	95.14 (14.92)	109.44 (13.08)
Motor milestones (months)	Sitting^a^	7.89 (5.31)	8.71 (7.72)	6.52 (1.73)
	Crawling^a^	7.65 (2.87)	10.27 (5.83)	8.29 (1.70)
	Standing up^a^	9.35 (2.15)	9.11 (2.15)	9.76 (1.66)
	Walking^a^	14.83 (3.94)	13.33 (2.57)	12.94 (2.16)
	%
Absence of a motor millestone^b^	Crawling	58.0%	32.0%	39.6%
Motor milestones delayed according to Haizea-Llevant Scale^c^	Sitting	8.3%	7.4%	5.2%
	Standing up	4.2%	1.9%	5.2%
	Walking	12.5%	3.7%	1.0%

Note: ELBW: Extremely low birth weight. VLBW: very low birth weight. VLBW: weigh ≤1,500 g at birth; ELBW: weigh ≤ 1,000 g at birth.

^a^ Preterm data are reported with corrected age.

^b^ The absence of crawling is considered a normal variant of development.

^c^ Delay was considered when there was an absence of sitting without support at 9 months, not standing up even with support at 12 months and the absence of autonomous walking at 16–18 months. All preterm children were labeled according to their corrected age.

**Table 2.** Perinatal factors for the ELBW and VLBW sample.

		Mean (SD)	Range [Min–Max]
Gestational age (days)		208.93 (19.73)	[171–255]
Birth weight (grams)		1151.04 (240.60)	[690–1475]
Cranial perimeter (centimeters)		26.21 (2.45)	[20–30]
Apgar score 5 (minutes)		8.46 (1.55)	[2–10]
Supplemental oxygen (days)		183.37 (321.86)	[0–1728]
Mechanical ventilation (days)		127.64 (227.42)	[0–192]
Stay in Neonatal Intensive Care Unit (NICU) (days)		42.66 (32.51)	[3–143]
		N (%)
Use of prenatal steroids		54 (67.1%)
Cesarean delivery		24 (31.6%)
Multiple birth		24 (31.6%)
Apnea		15 (19.7%)
Use of postnatal steroids		4 (5.3%)
Patent ductus arteriosus (PDA)		20 (26.3%)
Necrotizing enterocolitis (NEC)		3 (3.9%)
Retinopathy of prematurity (ROP)		4 (5.3%)
Intraventricular hemorrhage (IVH)	0	58 (76.3%)	
	I	12 (15.8%)	
	II	4 (5.3%)	
	III	2 (2.6%)	
	IV	0 (0.0%)	
Periventricular leukomalacia		3 (3.9%)

Note: ELBW: Extremely low birth weight. VLBW: very low birth weight.

### 2.2. Outcomes

The MABC-2-C [12] consists of a battery of motor tests and a checklist protocol designed to identify children aged 5 to 12 years who present motor difficulties. The checklist can be completed by parents or professionals who work directly with the children (teachers or therapists). In the present study, only the checklist was employed and only the parents filled it out. MABC-2 checklist comprised questions about the child's motor behavior in different everyday situations, such as in the classroom, during recreational and physical education activities and in personal care. The checklist is divided into three sections (A, B and C). Sections A and B describe the child's interactions with their environment, and Section C focuses on non-motor factors that can affect movement. In the current study, parents completed Sections A and B. Section A (MABC-2-C-A-Static, 15 items) evaluates the child's mobility in a static and predictable environment; it is subdivided into Personal Autonomy (A1), Classroom Abilities (A2) and Sport and Recreational Activities (A3). Section B (MABC-2-C-B-Dynamic) assesses the child's mobility in a dynamic and unpredictable environment; it is composed of a subsection of Personal Autonomy + Classroom Abilities (B1), Ball Skills (B2) and Sports and Recreational Activities (B3). In each item, the child's usual motor behavior is rated on a Likert scale. The items scores are added up to obtain a total score, to which a “traffic light” indication can be attached, i.e., a green light for scores close to the average, an amber light for at risk of suffering from movement problems and a red light for high probability of motor problems. For the present study, we considered all the scales and subscales: the MABC-2-C total score; the MABC-2-C A-Static, A1, A2 and A3; and the MABC-2-C B-Dynamic, B1, B2 and B3.

The Haizea-Llevant Scale [13] was applied as a screening instrument to evaluate the milestones of early motor development. Parents were asked about the following motor milestones and the age (in months) at which their child reached them: when they could sit up; when they could crawl, understanding it as any type of locomotor strategy considered as normal in development performed by the infant (crawling on hands and knees, stomach creeping, bottom shuffling, rolling, asymmetrical crawling, seal creeping, etc.) [14]; when they could stand up; and when they could walk by themselves. The Haizea-Llevant Scale assesses the level of development of children from 0 to 5 years of age, and it includes a range of ages for normal attainment of certain developmental milestones. It consists of 97 items, which assess development as follows: Socialization area (26 items), Language and Logical-Mathematical area (31 items), Postural area (21 items) and Handling area (19 items). The items employed in this study correspond to the Postural area. The absence of sitting without support at 9 months, not standing up even with support at 12 months and the absence of autonomous walking at 16–18 months were considered warning signs for postural control. The absence of crawling is considered a normal variant of development, that is, 18% of children do not crawl and this does not imply pathological development [15].

The RIST test was used as a screening method for IQ. It consists of two tasks: one for verbal IQ (Guess what) and another one for non-verbal IQ (Odd-item) assessment. Its purpose in the present study was to exclude those children, both preterm and controls, who scored 70 points or less, regarding them as having low cognitive performance that could potentially affect psychomotor development.

**Table 3.** Perinatal risk factors from preterm sample considered for their motor development.

Prenatal and maternal conditions	Single or multiple pregnancy Vaginal or Caesarean delivery Prescribed maternal corticoid

Early postnatal interventions and treatments	Apgar score at 5 minutes Gestational age Birth weight Cranial perimeter Days of supplemental oxygen Days of ventilation Postnatal steroids
Early postnatal diseases and pathologies	NEC PDA Apnea ROP Intraventricular hemorrhage (from Grades 0 to IV) Periventricular leukomalacia (PVL)

For the perinatal risk factors analysis, the variables were retrieved from medical records and selected from existing literature on motor development (Table 3). About the diagnoses of the following variables, all preterm infants underwent at least three cranial ultrasound scans from the first week of life, with different frequency depending on their gestational age. A magnetic resonance imaging (MRI) or computed tomography (CT) scan was performed before discharge in those cases with Grade III–IV IVH and/or periventricular leukomalacia (PVL). Regarding retinopathy of prematurity (ROP), its diagnosis involved a fundus examination from the fourth week of life (never before the 30th week of gestational age) until hospital discharge. Diagnosis of ROP was based on a fundus examination. It was performed by a specialized paediatric ophthalmologist. The frequency of examinations also depends on the gestational age and/or the pathology seen.

### 2.3. Procedure

The primary caregivers whose children fulfilled the inclusion criteria were informed by a letter of the purpose of the research, and they were given the opportunity to participate in the study. Those who accepted to participate in the study provided written informed consent before the study began. Then, a pediatrician interviewed the parents, who completed the questionnaires while the children performed the IQ test applied by a psychologist. The duration of the appointment was approximately one hour.

### 2.4. Statistical analysis

Analyses were performed using SPSS 19.0 for Windows. Means, standard deviations and percentages were calculated for descriptive data (sociodemographic variables, MABC-2 checklist, milestones and perinatal factors). The Chi-square test, with Cramer's V as a measure of effect size, was used to compare groups on the nominal variables. A Student's t-test was employed to compare the performances of the control group and the preterm children, and ANOVA with a Bonferroni post-hoc test was used to compare the ELBW, VLBW and control groups. Cohen's d for the Student's t-test and Eta-squared for the ANOVA were used to estimate the effect size. Both ANOVA and the Student's t-test were followed by an ANCOVA to control for participants' age and gender. Pearson correlation coefficients were calculated for the motor outcomes in preterm children and milestones and perinatal factors. Significantly correlated variables were included in a stepwise regression model. A p-value lower than 0.05 was considered significant. The data that support the findings of this study are available from the corresponding author upon reasonable request.

## 3. Results

### 3.1. Descriptive data

No significant differences were found between the preterm group and the control group in terms of age, sex, maternal education level or laterality, nor did the groups significantly differ in the age (corrected for the preterm group) at which they reached all motor milestones. However, some significant differences were found when comparing the ELBWs and VLBWs with the controls for the ages of crawling (F_2,87_ = 3.410, *p* = 0.038, η^2^ = 0.073) and walking (F_2,149_ = 4.318, *p* = 0.015, η^2^ = 0.055). Post-hoc comparisons revealed that such differences in walking were between the controls and ELBW children (*p* = 0.011), while the comparisons for crawling did not remain statistically significant.

We also found significant differences in the number of controls and preterm children who did not crawl (χ^2^_1_ = 6.443, *p* = 0.011, V = 0.193), although we did not find significant differences between control and preterm children regarding the number of children in each group who were delayed in the development of each milestone (*p* > 0.05).

### 3.2. Motor comparison

Starting with the risk classification proposed by the checklist, 19.31% of the entire preterm sample (N = 17/88) were identified as at risk of having motor difficulties: medium risk in 4.5% (N = 4) and high risk in 14.77% (N = 13). The differences between the percentage of preterm and the percentage of controls that were classified as at-risk were not statistically significant (p = 0.086). Regarding the birth weight, in the VLBWs, 2% (N = 1/54) were classified as at medium risk, and 18.4% as at high risk (N = 9/54); in the ELBWs, 13.6% were labeled as at moderate risk (N = 3/24), and 9.1% as at high risk (N = 2/24). In the control group, 12.5% of the sample reached risk values (N = 12/96), with 6.25% reaching moderate risk (N = 6), and 6.25% reaching high risk (N = 6). No significant differences were found between the risk percentages for the VLBWs and ELBWs (p = 0.715).

Significant differences were found between preterm children and controls in the MABC-2-C total score (t_160_ = −3.091; *p* = 0.002, d = 0.473), MABC-2-C A–Static (t_165_ = −3.754; *p* = 0.001, d = 0.559), MABC-2-C A1 (t_167_ = −3.917; *p* < 0.001, d = 0.578), MABC-2-C A2 (t_166_ = −2.963; *p* = 0.003, d = 0.446), MABC-2-C A3 (t_166_ = −2.576; *p* = 0.011, d = 0.390) and MABC-2-C B3 (t_184_ = −2.838; *p* = 0.005, d = 0.439). MABC-2-C B–Dynamic, B1 and B2 did not show significant differences. All of these comparisons resulted in low (0.3) to medium (0.5) effect sizes. These significant differences were adjusted for sex and age by applying ANCOVA (*p* > 0.05). Considering the preterm ELBWs, preterm VLBWs and controls, we obtained significant differences for MABC-2-C A – Static (F_2,166_ = 3.787; *p* = 0.025, η^2^ = 0.044) and MABC-2-C A1 (F_2,168_ = 5.276; *p* = 0.006, η^2^ = 0.059). Bonferroni's post-hoc analysis revealed that MABC-2-C A–Static differences were only between the ELBW and control groups (*p* = 0.036), whereas MABC-2-C A1 differences were obtained between the ELBWs and controls (*p* = 0.030), and between the VLBWs and controls (*p* = 0.034). These differences remained as statistically significant after controlling for age and gender in the ANCOVA.

### 3.3. Current motor outcomes and their relationship with early motor milestones in the preterm group

First, we calculated the Pearson correlations between the different measures of MABC-2-C and the birth weight of the preterm children. As we did not obtain any statistically significant correlation (*p* > 0.05), we considered the group of preterm children as a whole, without taking into account their birth weight, for the rest of the analyses. Thus, the correlation analysis yielded significant associations between the MABC-2-C total score and the corrected age of sitting (r = 0.527; *p* = 0.003), crawling (r = 0.664; *p* < 0.001) and walking (r = 0.326; *p* = 0.004). MABC-2-C A–Static was significantly associated with the age of sitting (r = 0.473; *p* = 0.007), crawling (r = 0.650; *p* < 0.001) and walking (r = 0.271; *p* = 0.007). MABC-2-C-B–Dynamic did not show any significant association with the milestones. Significantly correlated variables were included in a stepwise regression model, and only crawling was entered in the model, predicting the outcome of the MABC-2-C total score (R^2^ = 0.483).

### 3.4. Current motor outcomes and their relationship with perinatal risk factors

Correlation analyses were performed to identify significant perinatal variables with the scales of motor assessment. The results showed that the MABC-2-C total score was significantly correlated with the use of prenatal steroids (r = −0.299, *p* = 0.014), primary apnea (r = −0.294, *p*=0.016), days of ventilation (r = 0.290, *p* = 0.017) and intraventricular hemorrhage grade (r = 0.302, *p* = 0.013). MABC-2-C A–Static correlated significantly with the use of prenatal steroids (r = −0.271, *p* = 0.027), patent ductus arteriosus (r = −0.270, *p* = 0.027), apnea (r = −0.332, *p* = 0.006), days of ventilation (r = 0.342, *p* = 0.005) and intraventricular hemorrhage grade (r = 0.349, *p* = 0.004). MABC-2-C B–Dynamic did not show any association with perinatal variables. Significantly correlated variables were included in a stepwise regression model. Intraventricular hemorrhage, the use of prenatal steroids and the days of ventilation were entered in the model, predicting the outcome of the MABC-2-C total score (R^2^ = 0.227).

## 4. Discussion

The present study aimed to examine the psychomotor development of VLBW and ELBW school-aged preterm children, and to relate it to early aspects of their development. The ultimate goal was to propose a simple tool that allows health professionals in a regular consultation to screen the motor development of preterm.

First, preterm children scored lower than the control group on the MABC-2-C. With this tool, we identified almost 20% of preterm children at risk, similar to previous studies that employed this same checklist (23–36%) [16]. Only Part A of the checklist, which evaluates motor performance in a static environment, differentiated controls from preterm, while any significant difference was obtained in Part B, which assesses actions in a moving environment. Although such differences in Part A may be surprising because it assesses simple actions, regarding the factorial structure of the checklist proposed by other authors [17], many of the items in Part A fall into factors of gross motor skills, coordination, fine motor skills and balance; this coincides with some of the main motor limitations previously found in this population [2].

Considering birth weight, the ELBWs and VLBWs obtained lower scores than the controls on Scale A1, which is related to personal autonomy behaviors. Functional difficulties related to self-care have been previously reported in preterm children [18], as well as in DCD [19]. Interestingly, we did not find evidence of birth weight as a relevant factor when examining these motor activities.

In the preterm group in general, the age at which they achieved sitting, crawling and walking was related to their motor development in later childhood, but only the age of crawling was a significant predictor of such subsequent motor development. Previous research has found associations between motor milestones, i.e., grasping an object [20], walking [5] or acquiring fewer milestones at a given age [21], and later motor development in preterm children. Contrary to previous evidence [9], the preterm children in our sample did not suffer a delay in the acquisition of these motor milestones at the corrected age, highlighting milestone attaintment at an appropriate age may even be related to later motor development.

The age of crawling being a significant predictor of later motor development has some limitations. First, a high percentage of the preterm and term-born children in the sample did not crawl. Second, there were fewer preterm children who crawled than controls. Therefore, it is difficult to consider crawling as a "universal" predictor of later development, given that the absence of crawling is common within development; yet, if a preterm infant starts to crawl later, this may be a warning sign for subsequent motor development in infancy.

Later psychomotor development was associated with apnea, prenatal steroid use, days of mechanical ventilation and intraventricular hemorrhage, the latter three factors being significant predictors of later motor development. Coinciding with our findings, events involving early brain damage, such as intraventricular hemorrhage, pose a greater risk for poorer motor development in later childhood [2],[10],[22]. Factors affecting respiration, such as primary apnea and the requirement for mechanical ventilation, were related to later motor development. Previous studies have shown that bronchopulmonary dysplasia [2] and mechanical ventilation [23] are associated with poorer psychomotor outcomes in preterm children. These respiratory factors may cause certain brain alterations, manifesting as irregularities in the brain blood flow and brain oxygen supply [24]. Finally, according to our own results, previous studies also demonstrated that the use of steroids is associated with later motor development in preterm children [10],[25]. The prescription of prenatal steroids is used to prevent preterm delivery and promote lung maturation during gestation [26]. Therefore, it is highly probable that taking this medication does not in itself promote worse motor development, but rather that those fetuses that received the steroids already presented a higher risk of preterm birth and worse pulmonary maturation.

A limitation of our study is that our findings were obtained from the checklist and not from the complete MABC-2 evaluation. In this regard, there is a marked absence of studies that relate the checklist or questionnaire scores to performance-based tasks. Other questionnaires widely used for the diagnosis of DCD, such as the Developmental Disorder Coordination Questionnaire (DCDQ) [27], correlate with the MABC-2 battery [28], whereas, in other studies, this association is not found [29]. Besides, the MABC-2 checklist seems to have low sensitivity compared to the entire battery for the general child population [30] or children who had suffered from neonatal illness [31]. Some paradoxical results have even been found regarding this matter, particularly, the MABC-2 checklist results being inversely correlated with the DCDQ outcomes [29]. However, some other studies demonstrated that the MABC-2 checklist is strongly associated with performance-based tasks in terms of fine motor skills and hand coordination [32], and that the checklist has appropriate psychometric properties and good internal consistency, with a moderate association with other questionnaires and the entire battery, although its sensitivity remains low [17]. Considering all of these outcomes, the MABC-2 checklist could be used as a screening form to quickly identify some motor difficulties as long as the potential existence of some false-negative rates is taken into account.

## 5. Conclusions

In conclusion, ELBW and VLBW preterm children may present motor developmental disturbances in childhood, mainly those related to personal autonomy. The age of attainment of certain motor milestones in premature children may be related to later motor performance, without necessarily implying a delay in the age of acquisition. Likewise, certain perinatal factors related to early cerebrovascular events and respiratory difficulties are also associated with motor performance, probably because they involve an alteration in later brain development. In this sense, screening tools could be used for the detection of a possible case of motor developmental risk in regular pediatric consultations.
